# Room-temperature magnetocaloric performance in La_0.57_Nd_0.1_Sr_0.23_Ag_0.1_MnO_3_ manganite: a promising candidate for magnetic refrigeration

**DOI:** 10.1039/d6ra00854b

**Published:** 2026-05-20

**Authors:** H. Issaoui, M. Jeddi, N. Amri, F. Issaoui, E. Dhahri, E. K. Hlil

**Affiliations:** a Laboratory of Advanced Multifunctional Materials and Technological Applications, Faculty of Science and Technology of Sidi Bouzid, University Campus Agricultural City, University of Kairouan Sidi Bouzid 9100 Tunisia; b Applied Physics Laboratory, Faculty of Sciences, Sfax University BP 1171 3000 Tunisia; c Higher Institute of Computer Science and Multimedia, Gabes University BP 122 6033 Tunisia; d Institut Néel, CNRS – Université J. Fourier BP 166 38042 Grenoble France

## Abstract

The present paper presents an investigation of the structural, magnetic, magnetocaloric properties and critical behavior of La_0.57_Nd_0.1_Sr_0.23_Ag_0.1_MnO_3_ (LNSAMO) perovskite manganite, synthesized *via* solid-state reaction. X-ray diffraction confirms a rhombohedral structure with minor impurity phases. The FTIR spectra confirmed the formation of the structure of rhombohedral perovskite. Magnetization measurements reveal a sharp ferromagnetic to paramagnetic transition at a Curie temperature *T*_c_ ≈ 318 K. In the paramagnetic regime, the inverse susceptibility obeys the Curie–Weiss law with a positive Weiss temperature close to *T*_c_, indicative of strong ferromagnetic interactions. The magnetocaloric effect (MCE) was evaluated from isothermal magnetization curves using the Maxwell relation and Landau theory analysis, yielding a moderate maximum magnetic entropy change (−Δ*S*^max^_M_) and relative cooling power (RCP) under different magnetic fields. The nature of the magnetic phase transition was examined *via* Banerjee's criterion and universal curve, confirming its second-order character. Heat capacity measurements near *T*_c_ exhibit characteristic features consistent with a continuous magnetic phase transition. The critical behavior of the LNSAMO sample was analyzed using isothermal magnetization measurements near the transition temperature, employing methods such as the modified Arrott plot (MAP), Kouvel–Fisher (KF) technique, and critical isotherm analysis (CIA).

## Abstract 2

The present paper presents an investigation of the structural, magnetic, magnetocaloric properties and critical behavior of La_0.57_Nd_0.1_Sr_0.23_Ag_0.1_MnO_3_ (LNSAMO) perovskite manganite, synthesized *via* solid-state reaction.

## Graphical abstract

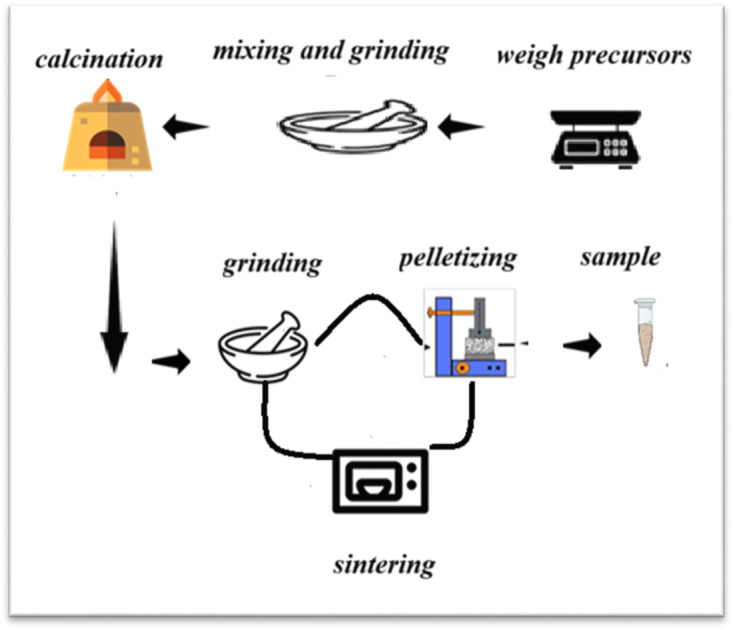

## 1 Introduction

In recent decades, perovskite manganites with the general formula R_1−*x*_A_*x*_MnO_3_, where R is a trivalent rare-earth ion (*e.g.*, La^3+^, Nd^3+^, Pr^3+^) and A is a divalent or monovalent cation (*e.g.*, Sr^2+^, Ca^2+^, Ag^+^), have attracted tremendous attention due to their rich variety of magnetic, electronic and thermal phenomena.^1–3^ Their magnetocaloric properties, which describe the material's response to an external magnetic field in terms of entropy and temperature change, have shown great promise for magnetic refrigeration applications.^4–6^ The magnetocaloric effect (MCE), especially near room temperature, is strongly influenced by the magnetic phase transition and the degree of magnetic ordering, both of which can be finely tuned by chemical substitution.

One of the most widely studied parent compounds is LaMnO_3_, which exhibits an antiferromagnetic insulating state due to strong Jahn–Teller distortion and cooperative orbital ordering.^7^ Upon partial substitution of La^3+^ with divalent ions such as Sr^2+^, the system undergoes a transition from antiferromagnetic insulating to ferromagnetic metallic behavior, accompanied by the emergence of double exchange interactions between Mn^3+^ and Mn^4+^ ions.^8,9^ This double exchange mechanism plays a key role in establishing long-range ferromagnetic order and a sharp magnetic phase transition, prerequisites for a large magnetocaloric response.^10^ Furthermore, partial substitution of La^3+^ by smaller rare-earth cations such as Nd^3+^ introduces size disorder and chemical pressure, modifying the Mn–O–Mn bond angle and reducing the bandwidth of eg electrons. This structural distortion can suppress the transition temperature and enhance magnetic fluctuations, often leading to broader entropy change peaks and improved refrigerant capacity.^11,12^ Additionally, the incorporation of Ag^+^ ions in the A-site lattice is of particular interest. Although monovalent, Ag^+^ may occupy interstitial or substitutional sites and act as an electron acceptor, further modifying the Mn valence balance and magnetic interactions. Previous reports have shown that Ag doping can enhance magnetic ordering, reduce resistivity, and improve thermal and magnetic stability.^13–15^

In this context, the compound La_0.57_Nd_0.1_Sr_0.23_Ag_0.1_MnO_3_ (LNSAMO) represents a strategically engineered manganite system combining cationic size mismatch (La/Nd), mixed valence states (La/Sr), and enhanced bond distortion/electronic bandwidth control (Ag). Such synergistic substitution is expected to tailor the magnetic interactions and generate a pronounced magnetocaloric effect near the Curie temperature (*T*_c_), making it a strong candidate for room-temperature magnetic refrigeration.

The main objective of this work is to investigate the structural, magnetic, magnetocaloric and critical behavior of the perovskite-type manganite compound La_0.57_Nd_0.1_Sr_0.23_Ag_0.1_MnO_3_ (LNSAMO), in order to assess its potential for magnetic refrigeration applications near room temperature.

## 2 Experimental procedure

**Fig. 1:**
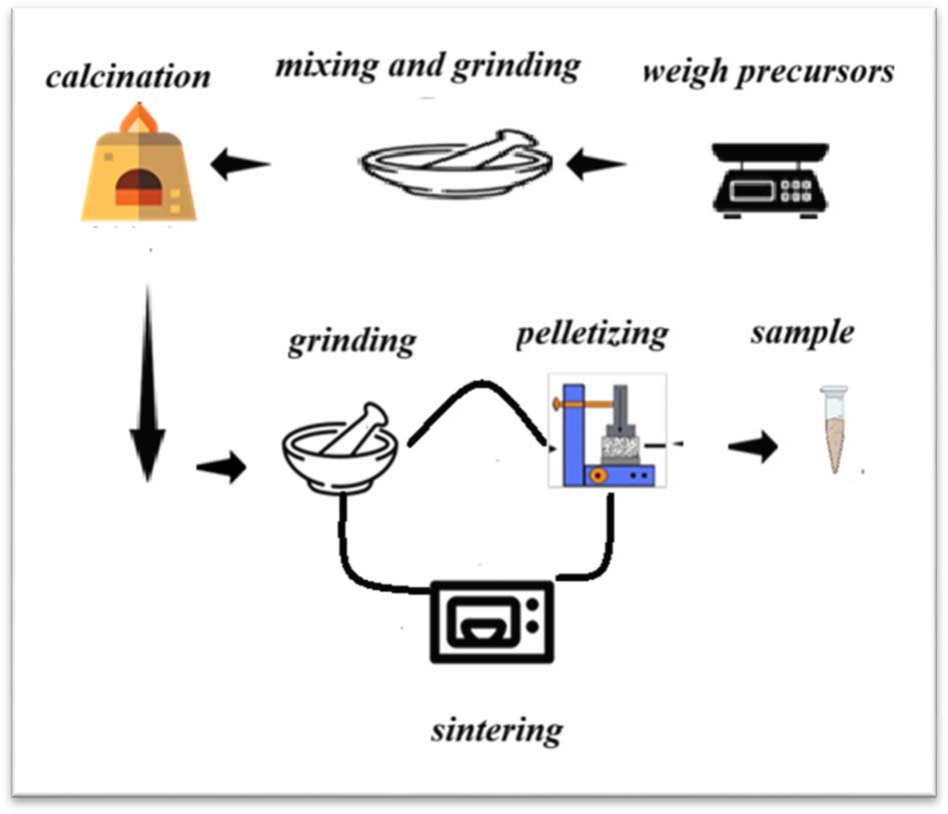
Schematic diagram representing the various synthesis steps for LNSACO sample using solid-state reaction.

Ceramic sample of La_0.57_Nd_0.1_Sr_0.23_Ag_0.1_MnO_3_ was synthesized using the standard solid state reaction method at high temperature, by mixing of La_2_O_3_ Sr_2_O_3_, Nd_2_O_3_, SrO, Ag_2_O and MnO_3_ (ref. 16) up to 99.9% purity in the desired proportions (Fig. 1). The precursors were preheated to *T* = 200 °C for 2 hours before weighing. These powders were then mixed in the required atomic ratio in an agate mortar and pressed, to reduce the particle size to the order of nonometric and give a homogeneous compound. The pellets have been heated under air at 1573 K for 13 hours,^17^ followed by slow cooling in the room temperature, the choice of this very high heat treatment temperature, to get rid of all impurities.

The X-ray diffraction data were carried out using a diffractometer equipped with a single crystalline graphite monochromator (MACMXP18 powder X-ray diffractometer). The diffraction pattern was collected with CuKa the radiation covers an overall range of 10 to 110 with a step of 0.015. Structural refinement has been performed out by the Full Prof program^18^. The measurement of magnetization was selected using a magnetometer (BS_2_) developed at the Néel Institute of CNRS Grenoble. Magnetization was measured as a function of field (*µ*_0_*H*) for different temperatures (*T*). The magnetic properties were carried out at a temperature 5 to 300 K in an applied magnetic field (*µ*_0_*H*) of 0.05 T.

## 3 Results and discussions

### 3.1 Structural characterization

**Fig. 2:**
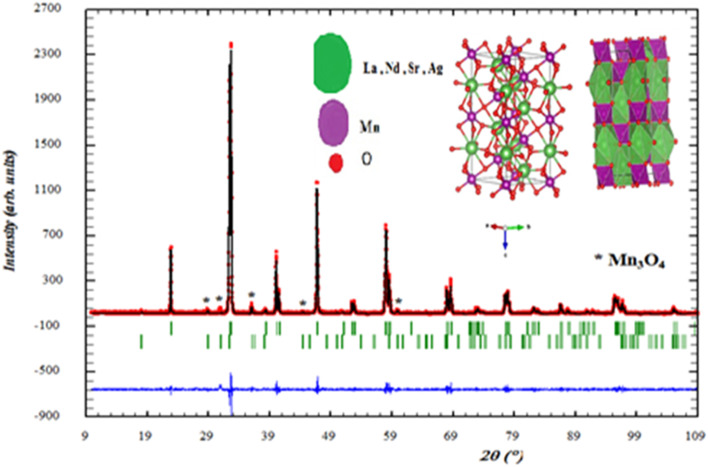
Refined X-ray diffraction pattern and crystal structure of LNSAMO compound (solid black circle represents the observed pattern, continuous red line represents computed pattern and the blue line represents distinction between the observed and computed patterns).

The crystal structure of the LNSAMO compound is analyzed using powder X-ray diffraction (XRD) at room temperature.^17^ The obtained diffractogram (Fig. 2) shows well-defined peaks indicating good crystallinity. All peaks are indexed in rhombohedral structure with *R*3̄*c* space group in positions 6a (0, 0, 1/4) for atoms (La, Nd, Sr, Ag), 6b (0, 0, 0) for atoms (Mn) and 18e (*x*, 0, 1/4) for oxygen. A minor secondary phase Mn_3_O_4_ is also detected. Such impurity phase suggests incomplete incorporation of Ag or Mn oxides into the perovskite lattice.^19^ The schematic representation of the LNSAMO crystal structure is depicted in Fig. 2. The corresponding Rietveld refinement results are summarized in Table 1.

**Table 1:** Refined structural parameters of LNSAMO sample

Compound	LNSAMO
Groupe d'espace	*R*3̄*C*
*a* (Å)	5.511711
*b* (Å)	5.511711
*c* (Å)	13.341709
Volume *V* (Å^3^)	58.501

**La, Nd, Sr, Ag (site 6c)**	
*x*	0.00000
*y*	0.00000
*z*	0.25000

**Mn (site 6b)**	
*x*	0.00000
*y*	0.00000
*z*	0.00000

**O (1) (site 18c)**	
*x*	0.45109
*y*	0.00000
*z*	0.25000
*a*_R_ (Å)	5.4684
*α*_R_ (Å)	60.5166
Mn–O (long) (Å)	2362
Mn–O (intermediate) (Å)	2038
Mn–O (short) (Å)	1863
〈*d*_Mn−o_〉 (Å)	2087
*Q*_2_ (Å)	0.705
*Q*_3_ (Å)	−0.121
*t*	0.975
*D*_sc_ (nm)	24.34
*R*_f_	2.78
*χ* ^2^	3.20

The rhombohedral structure results from the distortion of the ideal cubic structure ABO_3_ along the diagonal of the cube. It is a unit cell containing two ABO_3_ formula units. This structure is described using the hexagonal unit cell, whose parameters are *a*_h_ = *b*_h_ = *a*_c_√2 and *c*_h_ = 2*a*_c_√2, where *a*_c_ is the lattice parameter of the ideal perovskite.

The primitive rhombohedral lattice parameters *a*_R_ and *α*_R_ are related to those of the hexagonal unit cell by the following relations:1
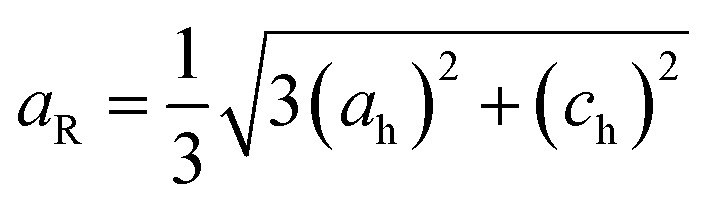
2
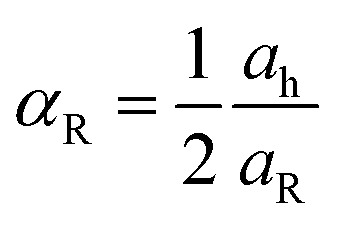


This structure can be described as a perovskite with two types of distortions relative to the cubic structure: a Jahn–Teller (J–T) distortion of the octahedron, originating from the presence of Mn^3+^ ions (3d^4^) in an octahedral crystal field; and a steric distortion.

**Fig. 3:**
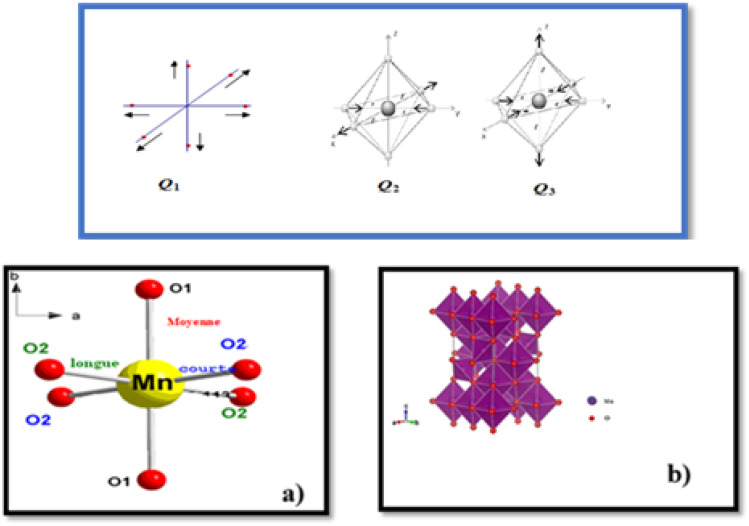
*Q*_2_ and *Q*_3_ represent vibration modes characterizing the distortion of MnO_6_ octahedra. Represents normal vibration mode *Q*_1_. (a) Manganese coordination polyhedron presented the distances; long medium and short in the basic plane and octahedral environment of the manganese atom. (b) Deformation of MnO_6_ octahedra belonging to the same system without taking into account rotations.

Indeed, the Jahn–Teller (J–T) distortion occurs through an anisotropic variation of the different bonds^20^. It is represented by the superposition of the two vibrational modes *Q*_2_ and *Q*_3_.^21,22^ The structure stabilizes by differentiating the three Mn–O bond lengths within the octahedron. The vibrational mode *Q*_1_ (Fig. 3) is not associated with the J–T distortion for the simple reason that it does not lift the degeneracy of the Mn ion's eg energy level, unlike the *Q*_2_ and *Q*_3_ vibrational modes. The values of the vibrational mode parameters *Q*_2_ and *Q*_3_ (Table 1), which correspond respectively to the distortion of the octahedron's basal plane and its elongation along the *c*-axis, can be calculated according to Kanamori^23^ using the following expressions:3
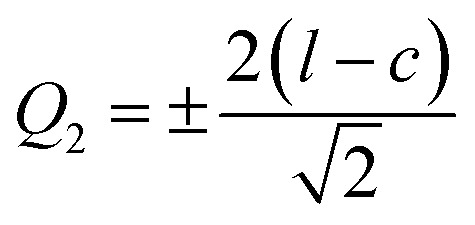
4
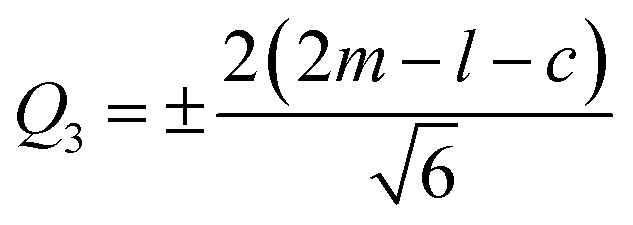
where *l* is the long Mn–O bond length,s is the short Mn–O bond length and *m* is the intermediate Mn–O bond length.

To confirm the existence of the perovskite-type structure and the degree of distortion of the LNSAMO compounds relative to the ideal cubic structure, we calculated the Goldschmidt tolerance factor^24^, as previously mentioned in the first chapter, using the following formula:5
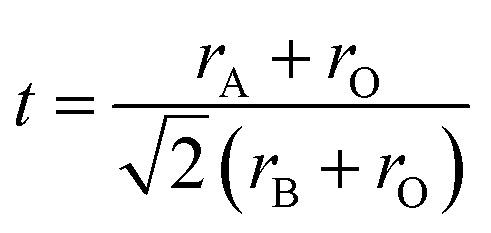
with *r*_A_ = *r*_(La,Nd,Sr,Ag)_, *r*_B_ = *r*_(Mn)_ and *r*_O_ are the ionic radii associated with the cations of sites A, B and oxygen, respectively.

In this context, the value of *t* for our LNSAMO sample is 0.975. This result is in agreement with the rhombohedral structure relative to the Rietveld refinement^17,25^.

The average crystallite size *D*_sc_ was estimated using the Debye–Scherrer formula:^26^6
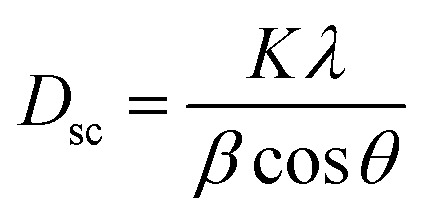
where *λ* = 1.5406 is the wavelength of CuKα radiation, *K* = 0.9 is the shape factor, *β* is the full-width at half-maximum of an XRD peak in radians and *θ* is the Bragg angle.

The calculated crystallite size is found to be approximately *D*_sc_ ≈ 24.34 nm, indicating a nanostructured material.

**Fig. 4:**
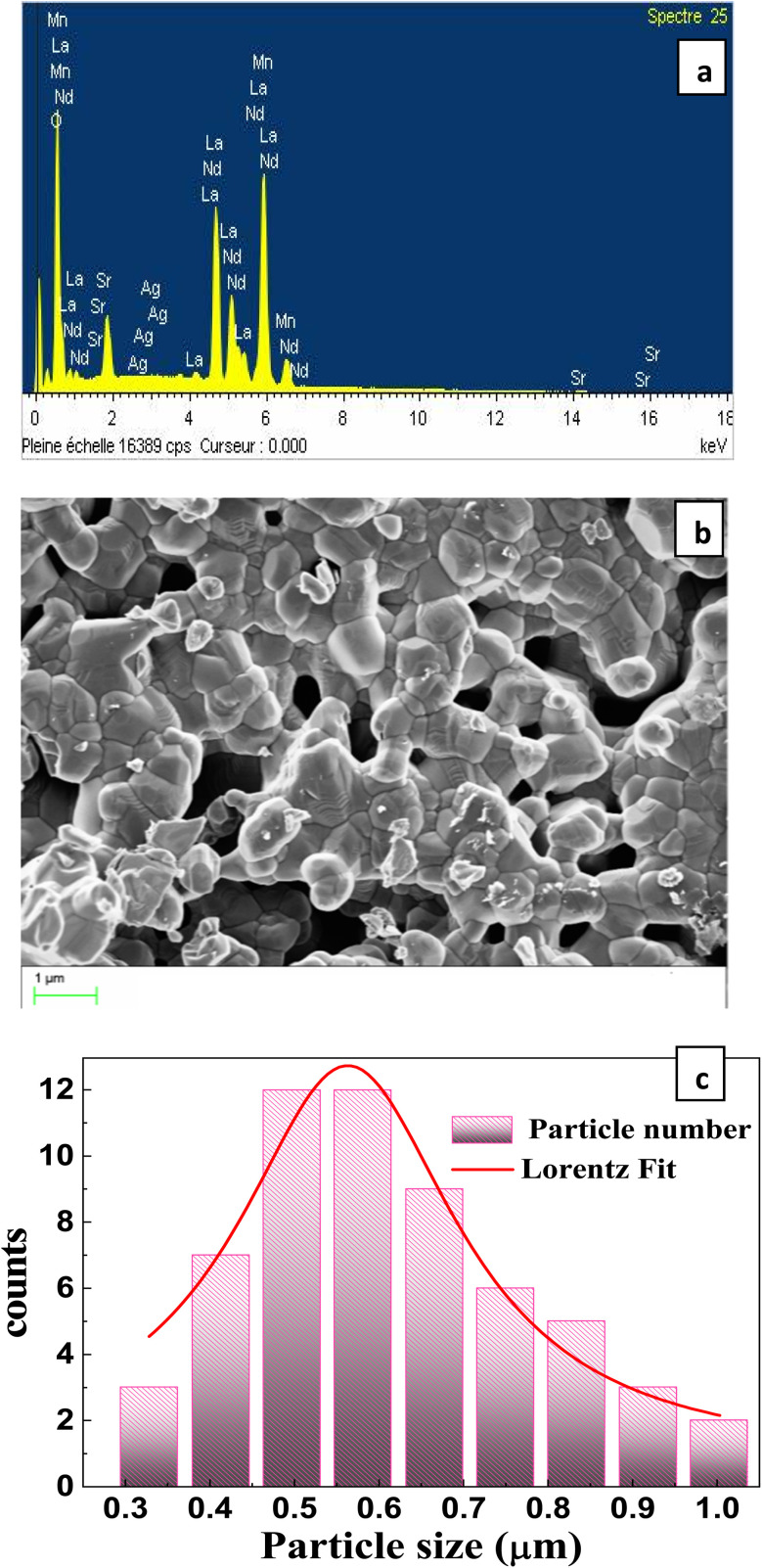
(a) EDX spectra, (b) SEM image of surface morphology and (c) particule size distribution of the LNSAMO sample.

To assess the homogeneity of cation distribution in LNSAMO compound, EDS analysis is performed on selected regions of the SEM image (Fig. 4a). The spectrum confirms the presence of La, Nd, Sr, Ag, Mn, and O, consistent with the nominal stoichiometry. No detectable foreign elements are observed, confirming the purity of the synthesized compound.

The surface morphology of the LNSAMO compound is examined by SEM (Fig. 4b). The micrograph reveals a relatively uniform grain distribution. Particles appear slightly agglomerated. To analyze the grain size distribution, ImageJ software is used. A histogram of the particle size distribution (Fig. 4c)reveals that the average grain diameter is approximately *D*_SEM_ ≈ 0.504 µm. The discrepancy between *D*_SEM_ and *D*_sc_ indicates that individual grains are aggregates of multiple crystallites.

### 3.2 FTIR spectroscopy

**Fig. 5:**
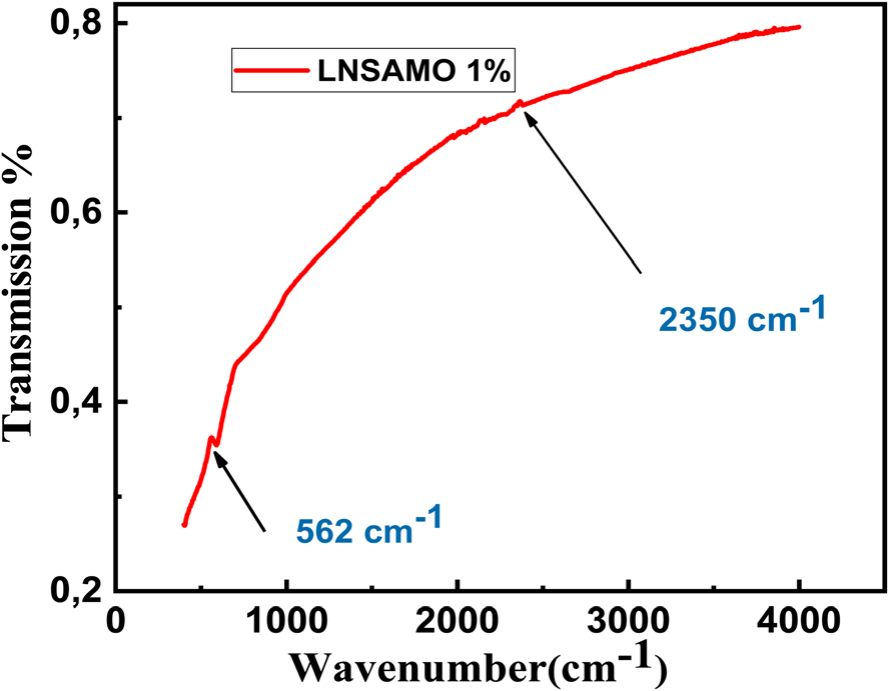
FTIR spectrum of LNSAMO manganite material.

The FTIR (Fourier Transform Infrared) spectrum provides valuable insight into the vibrational modes of ions within the crystal lattice. Fig. 5 displays the FTIR spectrum of the LNSAMO compound, recorded at room temperature over the wave number range of 500 to 4000 cm^−1^.

Two distinct absorption bands are observed, located at approximately 584 cm^−1^ and 2350 cm^−1^.

• The band near 584 cm^−1^ is attributed to the stretching vibrations of Mn–O bonds, which reflect internal structural changes, particularly in the bond length. This band may also involve bending (or folding) modes, which are sensitive to variations in the Mn–O–Mn bond angle.^27–31^ These vibrations are characteristic of the MnO_6_ octahedral environment, indicating localized vibrational modes within this structural unit.

• The band around 2350 cm^−1^ is typically assigned to the vibrational stretching and bending modes of adsorbed water molecules (OH) on the material's surface.^32^

### 3.3 Magnetic properties study

**Fig. 6:**
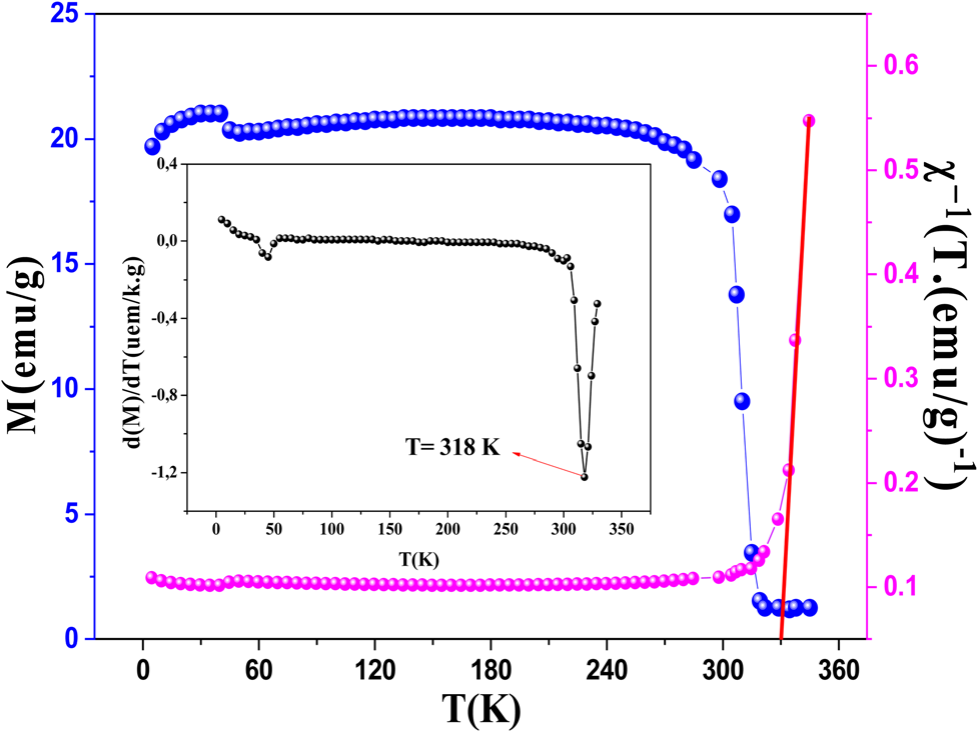
Variation of the magnetization and the inverse of the susceptibility as a function of temperature at 0.05 T of LNSAMO sample. The inset is the plot of d*M*/d*T versus T*.

The temperature dependence of the magnetization *M*(*T*), is measured under an applied magnetic field of 0.05 Tin the temperature range of 5 to 350 K. As shown in Fig. 6, the magnetization of LNSAMO exhibits a sharp increase upon cooling, indicating a transition from the paramagnetic (PM) to the ferromagnetic (FM) state. The Curie temperature *T*_c_, obtained from the minimum of d*M*/d*T*, is found to be approximately 318 K.

In the paramagnetic region, above the Curie temperature *T*_c_, the inverse magnetic susceptibility *χ*^−1^ (*T*) of the material follows the Curie–Weiss law, expressed as:^33^7
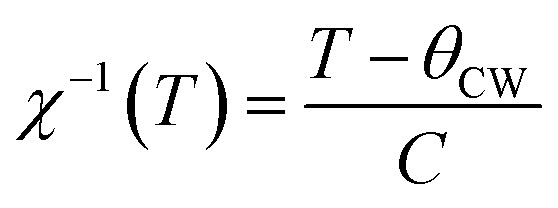
where *θ*_cw_ is Curie Weiss temperature and *C* is Curie constant.

By plotting the inverse susceptibility *χ*^−1^ as a function of temperature (Fig. 6), a linear behavior is observed in the high-temperature region, confirming the applicability of the Curie–Weiss law. From the linear fit, the Curie constant *C* and Curie–Weiss temperature *θ*_cw_ are extracted. The positive Curie–Weiss temperature *θ*_cw_ ≈ 330 K relatively close to *T*_c_ indicates dominant ferromagnetic interactions between Mn ions in LNSAMO.^34^ The slight difference between *θ*_cw_ and *T*_c_ can be attributed to the presence of spin fluctuations above *T*_c_.^35^

The Curie constant *C* is related to the effective magnetic moment *µ*_eff_ of the magnetic ions by the relation:^36^8
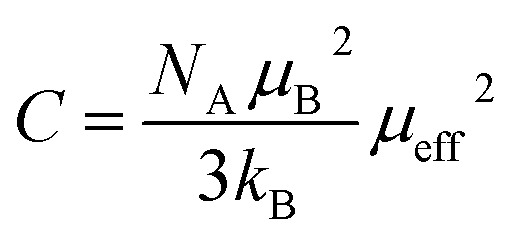
where *N*_A_ is the Avogadro number, *µ*_B_ is the Bohr magneton and *k*_B_ is the Boltzmann constant.

The effective magnetic moment *µ*_eff_, calculated from the Curie constant, is found to be 4.6 *µ*_B_, consistent with the mixed valence state of Mn ions.

### 3.4 Magnetocaloric effect

**Fig. 7:**
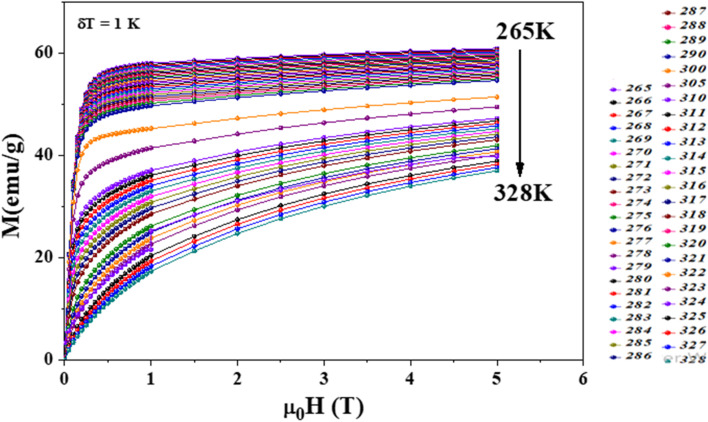
Isothermal magnetization curves measured at different temperatures around *T*_c_ for LNSAMO compound.

The isothermal magnetization *M*(*µ*_0_*H*, *T*) curves recorded at various temperatures (Fig. 7) display typical ferromagnetic behavior below *T*_c_.^37^

The magnetocaloric effect (MCE) in LNSAMO is investigated using isothermal magnetization measurements in the temperature range of 200 to 328 K and magnetic fields up to 10 T. The magnetic entropy change (Δ*S*_M_), which quantifies the MCE, is calculated from the isothermal magnetization curves using the Maxwell relation:^38^9
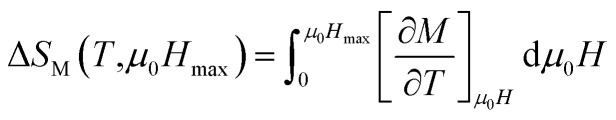


**Fig. 8:**
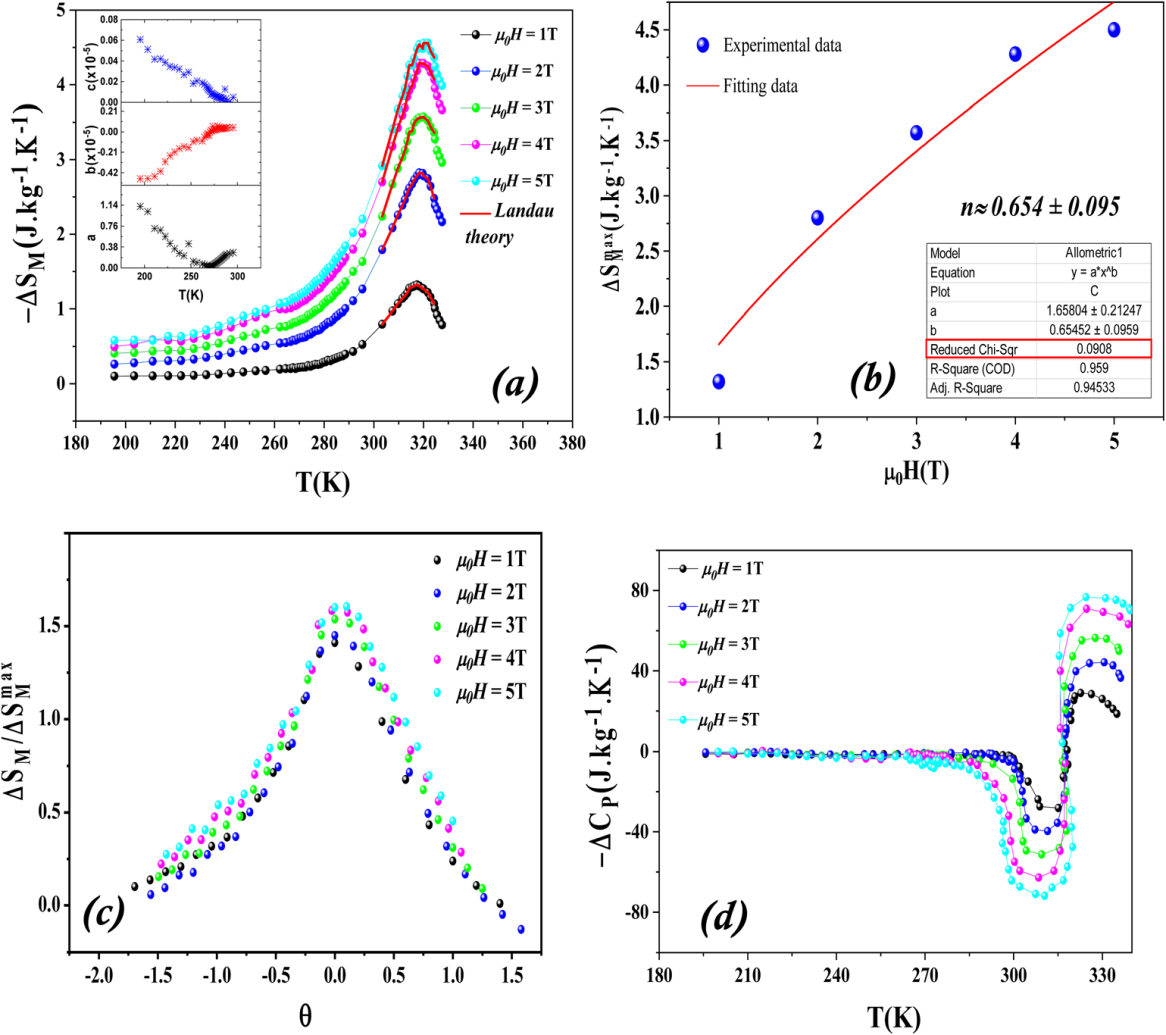
(a) Experimental and theoretical magnetic entropy changes under applied fields ranging from 1 to 5 T. The inset shows the temperature dependence of Landau's coefficients. (b) Variation of Δ*S*^Max^_M_*vs. µ*_0_*H*. (c) Universal curve and (d) the specific heat changes Δ*C*_P_ as a function of temperature for LNSAMO sample.

The temperature dependence of the magnetic entropy change−Δ*S*_M_(*T*) curves of LNSAMO compound are plotted in Fig. 8a. The maximum magnetic entropy change (−Δ*S*^max^_M_) occurs near the Curie temperature *T*_c_, where magnetic ordering changes rapidly. The maximum values of the magnetic entropy (−Δ*S*^max^_M_) are 2.81 and 4.52 J kg^−1^ K^−1^ under an applied magnetic field of 2 and 5, respectively. These values correspond to about 51 and 44% of those observed in pure Gd, respectively.^39,40^ Although these values are lower than those of pure Gd, they are comparable to many substituted manganite systems reported in the literature^41–43^. The moderate entropy change is characteristic of second-order magnetic phase transitions, which are generally associated with negligible magnetic hysteresis and good reversibility.

According to Oesterreicher and Parker^44^ the field dependence of the magnetic entropy change Δ*S*_M_ follows a field dependence power law:

10 Δ*S*_M_ ∝ (*µ*_0_*H*)^*n*^

The exponent *n*, characteristic of magnetic ordering, is found by fitting (Δ*S*_M_*vs. µ*_0_*H*) data (Fig. 8b), yielding *n* = 0.654. This result is in close agreement with the theoretical value of *n* = 2/3 predicted by the mean-field model for second-order magnetic transitions.^45^ The slight deviation from the ideal value is attributed to local magnetic in homogeneities within the sample.

Depending on the magnitude of (−Δ*S*_M_) and its full-width at half maximum (*δT*_FWHM_), the magnetocaloric efficiency can be evaluated through the relative cooling power (RCP).^46^ The latter, defined as the heat transfer between the hot and the cold sinks in one ideal refrigeration cycle, can be described by the following formula:

11 RCP = (−Δ*S*^max^_M_) × *δT*_FWHM_

The calculated RCP is 31.36 J kg^−1^ for *µ*_0_*H* = 2 T and 101.73 J kg^−1^ for *µ*_0_*H* = 5 T, which stands for about 64 and 25% of that observed in pure Gd, respectively.

To assess the applicability of our compound as magnetic refrigerant, the obtained values of (−Δ*S*^max^_M_) and RCP in our study are summarized in Table 2. These values are consistent with those reported for similar perovskite manganites operating near room temperature.^41–43^

**Table 2:** Summary of magnetocaloric properties of LNSAMO sample

Compound	*µ*_0_ *H* (*T*)	*T*_c_ (K)	−Δ*S*^max^_M_ (J kg^−1^ K^−1^)	RCP (J kg^−1^)	Ref.
La_0.57_Nd_0.1_Sr_0.23_Ag_0.1_MnO_3_ (LNSAMO)	1	318	1.32	31.36	Present work
	2		2.81	49.01	
	3		3.57	68.47	
	4		4.28	81.92	
	5		4.52	101.73	
Gd	2	—	5.5	164	38
	5		10.2	410	39
Nd_0.6_Sr_0.4_MnO_3_	5	275	3.594	202.054	40
Nd_0.6_Sr_0.3_K_0.1_MnO_3_	3	230	2.07	102.4	41
La_0.6_Ca_0.3_Sr_0.1_MnO_3_	2	304	2.89	98.17	42

In addition to its moderate magnetocaloric response, the present compound offers potential advantages such as chemical stability, relatively low material cost compared to rare-earth-based refrigerants, and the absence of thermal or magnetic hysteresis, which are desirable for practical magnetic refrigeration applications.

To further understand the magnetocaloric behavior, the experimental data are analyzed using the Landau phenomenological theory of phase transitions. According to Landau theory, the Gibbs free energy *G* near the magnetic transition can be expanded as a power series of the magnetization *M*:^47^12
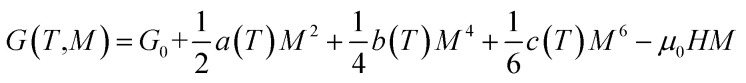
*a*(*T*), *b*(*T*) and *c*(*T*), the Landau coefficients, are shown in the inset of Fig. 8a.^48^

The temperature dependence of the Landau coefficients *a*(*T*), *b*(*T*), and *c*(*T*) extracted from the Arrott plot fitting is summarized in Table 3 for the critical temperature region.

**Table 3:** Temperature dependence of Landau coefficients near *T*_c_

*T* (K)	*a*(*T*)	*b*(*T*) (×10^−6^)	*c*(*T*) (×10^−10^)
289	−0.00599	5.02	−5.44
295	−0.00199	3.49	−4.40
298	−6.25 × 10^−4^	2.93	−3.88
301	1.62 × 10^−4^	2.72	−3.71
307	0.00196	1.88	−2.87
313	0.00295	1.47	−2.65
319	0.00376	0.967	−1.86
325	0.00423	0.685	−1.15

The magnetic entropy change (Δ*S*_M_) can be derived from the temperature derivative of the Gibbs free energy at constant field:^49^13
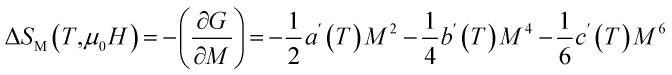
where *a*′(*T*), *b*′(*T*) and *c*′(*T*) are the temperature derivatives of the landau's parameters.

Fig. 8a compares the magnetic entropy change (Δ*S*_M_) obtained experimentally from the Maxwell relation with that calculated from the Landau theory. The good agreement between the two methods confirms the validity of the Landau theory in studying the magnetocaloric behavior of LNSAMO. The slight deviation of the Landau-derived entropy change close to *T*_c_ can be attributed to critical spin fluctuations and short-range magnetic correlations, which are not completely described within the mean-field framework of Landau theory.

To better understand the nature of the magnetic phase transition, the universal curve method was applied to the magnetic entropy change data.^50^ This approach evaluates whether the Δ*S*_M_(*T*, *µ*_0_*H*) curves obtained under different magnetic fields can be scaled onto a single, universal curve, a behavior that typically confirms a second-order magnetic phase transition. Each Δ*S*_M_ curve was first normalized by its maximum value (Δ*S*^max^_M_), and the temperature axis was rescaled using the reduced variable *θ*, which reflects the relative position of the temperature with respect to the Curie temperature *T*_c_:14
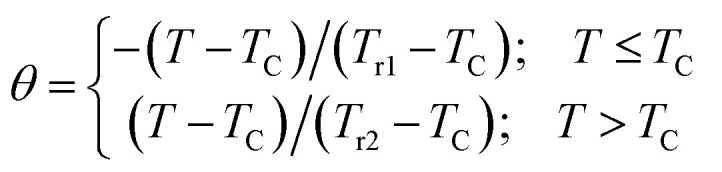
Here, *T*_*r*1_ and *T*_*r*2_ are two reference temperatures where the magnetic entropy is equal to half of the maximum value.

As shown in Fig. 8c, after this rescaling, all the curves collapse into one. This confirms that the magnetic phase transition in our material is of second order, which agrees with the results from the Arrott plots and Banerjee's criterion.

The heat capacity (Δ*C*_p_) of LNSAMO was measured under different applied magnetic fields. The magnetic contribution was extracted after subtracting the phonon background using a smooth baseline fitted well away from the transition region. Near the Curie temperature *T*_c_, Δ*C*_p_ exhibits two peaks of opposite sign: a positive peak just below *T*_c_ and a negative peak just above *T*_c_ (Fig. 8d). This sign change reflects the fundamental nature of the magnetic phase transition. Below *T*_c_, the system absorbs energy as it transitions from an ordered ferromagnetic state to a more disordered paramagnetic state, producing the positive peak. Above *T*_c_, the applied magnetic field aligns spins, reducing magnetic entropy and releasing energy, which results in the negative peak. Such behavior is characteristic of second-order magnetic phase transitions with continuous changes in magnetic ordering.^51^

### 3.5 Critical behavior study

**Fig. 9:**
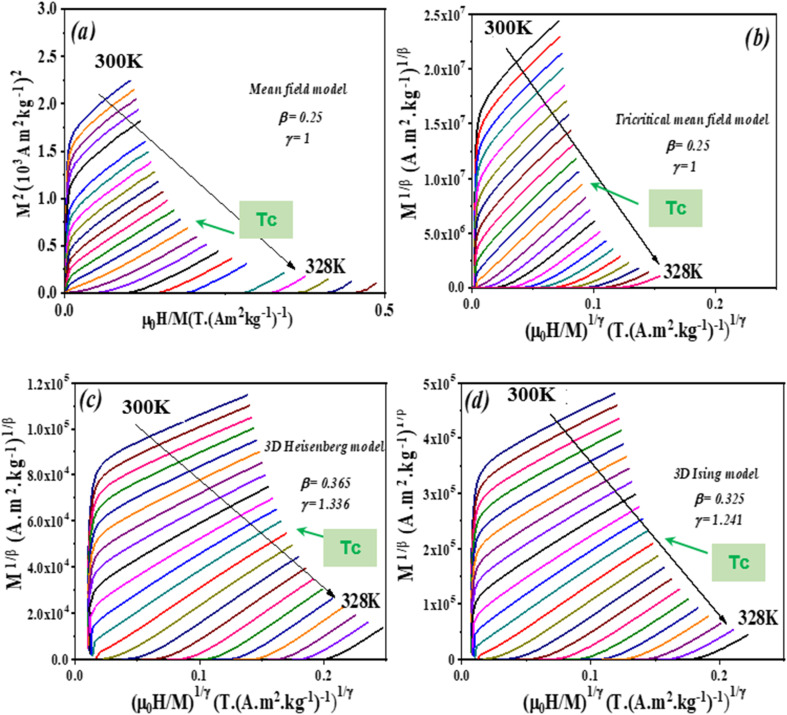
Modified Arrott plots (MAP): 
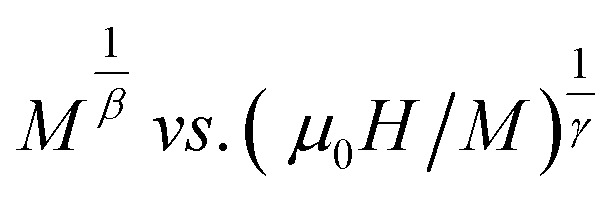
 for LNSAMO sample with mean field (a), tri-critical mean field model (b), 3D Heisenberg model (c) and 3D Ising model (d).

Fig. 9adisplays the Arrott plots, expressed as (*M*^2^*vs. µ*_0_*H*/*M*), obtained from the isothermal magnetization measurements. For all temperature values, the curves exhibit a positive slope, confirming that the magnetic phase transition from the ferromagnetic (FM) to the paramagnetic (PM) state is of second order. This behavior is consistent with the Banerjee criteria.^52^

The scaling theory describes a second-order magnetic phase transition that occurs at the Curie temperature *T*_c_ as a series of mutually dependent critical exponents. These exponents are calculated from magnetisation data using the asymptotic power-law relations listed below:^53^

15 *M*_S_(*T* < *T*_c_, *µ*_0_*H* → 0) = *M*_0_|*ε*|^*β*^

16
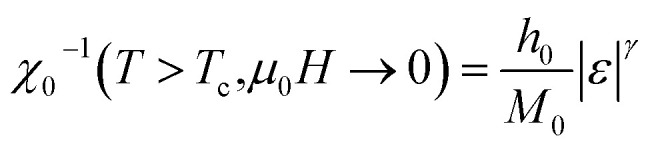
17
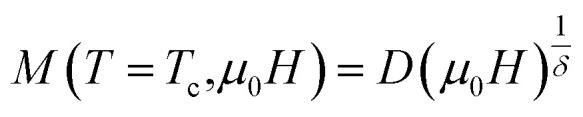
where 
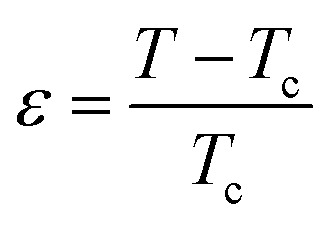
 denotes the reduced temperature. The parameters *M*_0_, 
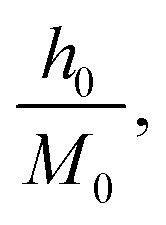
 and *D* are critical amplitudes. The exponent *β* characterizes the temperature dependence of the spontaneous magnetization *M*_S_ below *T*_c_, *γ* describes the behavior of the inverse magnetic susceptibility *χ*_0_^−1^ above *T*_c_, and *δ* governs the critical isotherm at *T*_c_.

The magnetic behavior near *T*_c_ can be divided into four theoretical models based on the values of these critical exponents (Table 4). To identify the model that best represents the magnetic interactions in the studied system, several analytical approaches are frequently used.

**Table 4:** Values of the critical exponents of LNSAMO compound

Model/compound	Technique	*T*_c_ (K)	*β*	*γ*	*δ*
Mean field model			0.5	1	3
Tri-critical mean field model			0.25	1	5
3D Heisenberg model			0.365	1.336	4.80
3D Ising model			0.325	1.240	4.82
La_0.57_Nd_0.1_Sr_0.23_Ag_0.1_MnO_3_ (LNSAMO)	MAP	218.210 ± 0015.1	0.319 ± 0.021	1.234 ± 0.002	
	KF	218.24 ± 0.009	0.322 ± 0.001	1.234 ± 0.003	
	CIA (exp.)				4.33 ± 0.051
	CIA (cal.)				4.87

According to the Modified Arrott–Plot (MAP) method, the data are analyzed based on the Arrott–Noakes equation:^54^18
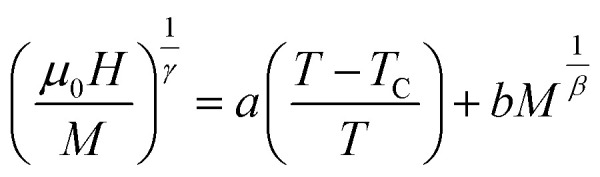
where *a* and *b* coefficients are considered to be constants.

To attempt the adequate model, the 
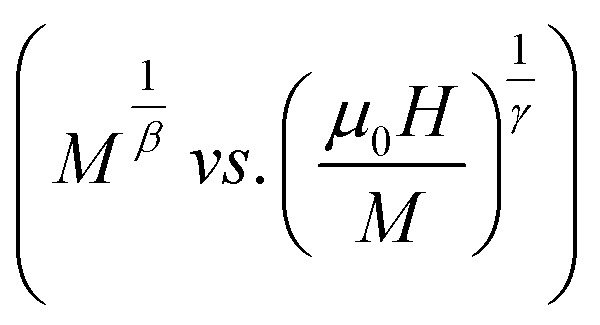
 plots should yield a set of reasonably good parallel straight lines.

Fig. 9 points out that, in the high field region, all models present nearly straight and parallel lines. Then, it seems difficult to specify which model is the most appropriate one to analyze the critical behavior of LNSAMO compound.

The relative slope (RS) is calculated in order to identify the most appropriate theoretical model. The RS parameter is defined at the critical region as:19
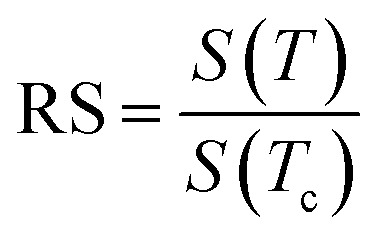
where *S*(*T*) and *S*(*T*_c_) are the slopes calculated from (MAP) around and at *T*_c_, respectively.

The model that best describes the critical behavior is the one for which the (RS *vs. T*) curve remains closest to unity over the studied temperature range.^55^

**Fig. 10:**
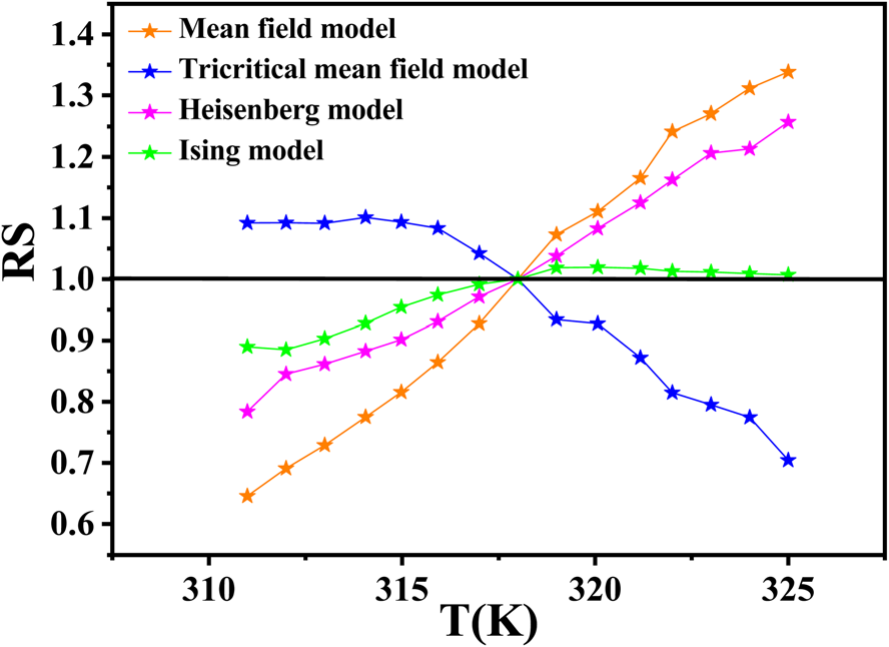
Relative slope (RS) as a function of temperature for LNSAMO sample.

As shown in Fig. 10, the 3D Ising model, with critical exponents *β* = 0.325 and *γ* = 1.240, gives the best agreement with the experimental data, indicating that it is the most suitable model for describing the critical behavior of the LNSAMO compound.

The observed 3D Ising critical behavior, which is associated with short-range magnetic interactions, can be correlated with structural distortions arising from the deviation of the Goldschmidt tolerance factor. Such a deviation induces tilting and distortion of the MnO_6_ octahedra, leading to variations in Mn–O–Mn bond angles and bond lengths. These structural modifications reduce the effective bandwidth and weaken long-range double–exchange interactions, thereby enhancing localized magnetic interactions and short-range magnetic order.

**Fig. 11:**
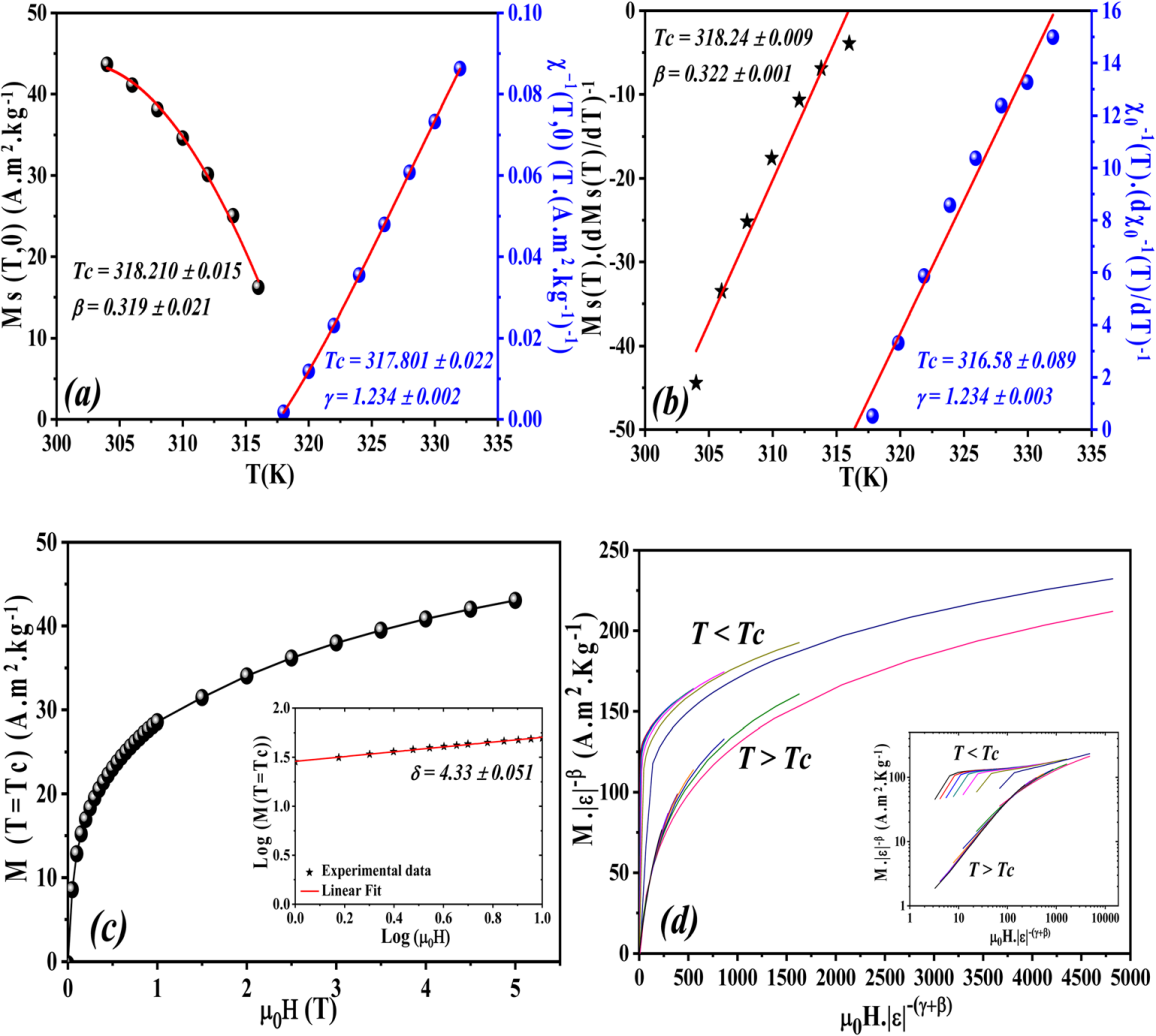
(a) Temperature dependence of spontaneous magnetization *M*_S_(*T*) and inverse initial susceptibility *χ*_0_^−1^(*T*). (b) Kouvel–Fisher (KF) plots for *M*_S_(*T*) and *χ*_0_^−1^(*T*). (c) Critical isotherm (*M vs. µ*_0_*H*). The inset exhibits the same curve on log–log scale and (d) Scaling plots *M*|*ε*|^−*β*^*vs. µ*_0_*H*|*ε*|^−*β*−*γ*^ below and above *T*_c_ for LNSAMO sample. The inset exhibits the same curve on log–log scale.

Following the MAP procedure, the spontaneous magnetization *M*_S_ and the inverse magnetic susceptibility *χ*_0_^−1^ are obtained by extrapolating the high-field linear regions of the isothermal magnetization curves to the intercepts on the 
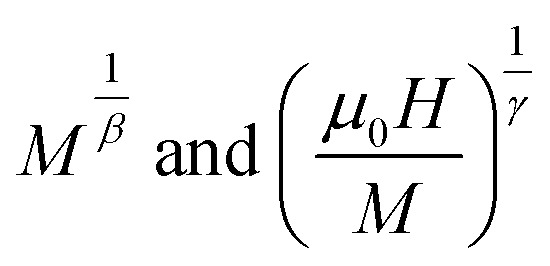
 axes, respectively. The temperature dependences of *M*_S_(*T*) and *χ*_0_^−1^(*T*) are presented in Fig. 11a. The fitting of these curves using eqn (15) and (16) allows the determination of the critical exponents *β* and *γ*, as well as the Curie temperature *T*_c_. The obtained values are summarized in Table 4.

According to the Kouvel–Fisher (KF) method,^56^ the *β*, *γ* and *T*_c_ values are accurately defined by constructing the following functions:20
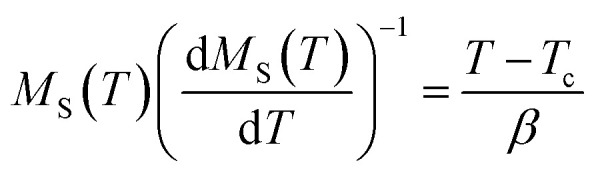
21
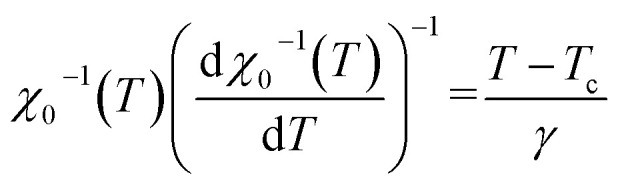


The 
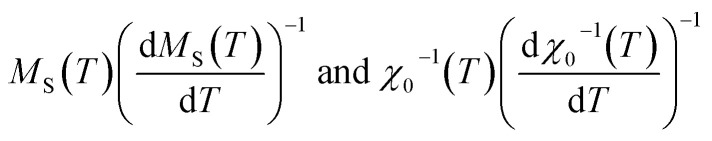
 plots should produce straight lines with slopes of 1/*β* and 1/*γ*. The Curie temperature *T*_c_ is obtained from the intercept of the linear extrapolation with the temperature axis, as shown in Fig. 11b. The critical exponents determined using the Kouvel–Fisher (KF) method are in good agreement with those obtained from the modified Arrott plot (MAP) method (Table 4). This agreement indicates that both methods are suitable for describing the critical behavior of the system.

Fig. 11c presents the critical isotherm (*M vs. µ*_0_*H*) curve, plotted on a log–log scale, at *T*_c_ = 318 K for LNSAMO compound. By fitting the experimental data using eqn (17), the critical exponent *δ* is obtained and the resulting value is listed in Table 4. This value is close to that predicted by the Widom scaling relation:^57^22
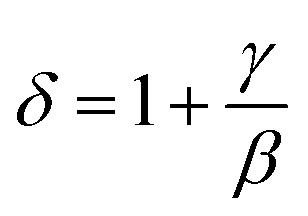


The findings indicate the accuracy of the obtained *β* and *γ* values.

The reliability of the obtained critical exponents can also be checked using scaling theory near the Curie temperature. In this region, the magnetization is described by:23
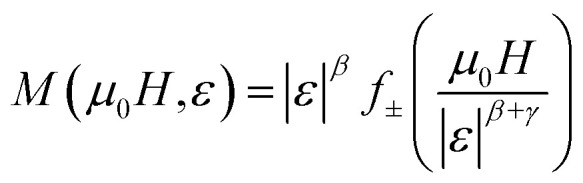
where *f*_+_ (*T* > *T*_c_) and *f*_−_ (*T* < *T*_c_) are regular analytic functions.

Fig. 11d shows the plot of (*M*|*ε*|^−*β*^*vs. µ*_0_*H*|*ε*|^−*β*−*γ*^) using the values of *β*, *γ* and *T*_c_ obtained from the (KF) method. The inset presents the same data in a log–log scale. All experiment points collapse into two branches, one for *T* < *T*_c_ and the other for *T* > *T*_c_. This scaling behavior confirms that eqn (23) is satisfied over the full range of the reduced variables, demonstrating the consistency and reliability of the extracted critical exponents.

## 4 Conclusion

In summary, the perovskite manganite LNSAMO was successfully synthesized using the solid-state method and investigated for its magnetic and magnetocaloric properties. Magnetization measurements revealed a clear ferromagnetic–paramagnetic transition near 318 K, making this material suitable for near-room-temperature applications. The Curie–Weiss analysis confirmed dominant ferromagnetic interactions. The magnetocaloric effect, evaluated from isothermal magnetization data and landau theory, showed a moderate magnetic entropy change and a relatively high RCP, comparable to other manganite-based materials. Arrott plots and Banerjee's criterion confirmed the second-order nature of the magnetic phase transition, further supported by the construction of a universal scaling curve for the normalized entropy change. Heat capacity measurements displayed typical features associated with continuous magnetic transitions. The values of the critical exponents *β*, *γ*, and *δ* are determined by analyzing the isothermal magnetization around *T*_c_ using the MAP method, KF method, and critical isotherm (CIA) method. The values agree with those of 3D Ising-like ferromagnets with short-range interactions. The scaling analysis confirms the validity of the critical exponents. Overall, these results demonstrate that LNSAMO exhibits magnetic and magnetocaloric properties suitable for practical magnetic refrigeration devices operating near room temperature.

## Conflicts of interest

There are no conflicts to declare.

## Data Availability

All data supporting this study have been included in the main article.
